# Detection, Emission Estimation and Risk Prediction of Forest Fires in China Using Satellite Sensors and Simulation Models in the Past Three Decades—An Overview

**DOI:** 10.3390/ijerph8083156

**Published:** 2011-07-28

**Authors:** Jia-Hua Zhang, Feng-Mei Yao, Cheng Liu, Li-Min Yang, Vijendra K. Boken

**Affiliations:** 1 Chinese Academy of Meteorological Sciences, 46 Zhongguancun Nandajie, Beijing 100081, China; 2 Center for Earth Observation and Digital Earth, Chinese Academy of Sciences, 9 Dengzhuang South Road, Beijing 100094, China; 3 College of Earth Sciences, The Graduate University of Chinese Academy of Sciences, 19 Yuquan Road, Beijing 100049, China; 4 National Satellite Meteorological Center, 46 Zhongguancun Nandajie, Beijing 100081, China; E-Mail: liucheng@nsmc.cma.gov.cn; 5 U.S. Geological Survey, Center for Earth Resources Observation and Science, Sioux Falls, SD 57198, USA; E-Mail: Lyang0117@yahoo.com; 6 Department of Geography and Earth Science, University of Nebraska at Kearney, 905 West 25th Street, Kearney, NE 68849, USA; E-Mail: bokenv1@unk.edu

**Keywords:** forest fire detection, fire emission estimation, forest fire risk model, satellite remote sensing, China

## Abstract

Forest fires have major impact on ecosystems and greatly impact the amount of greenhouse gases and aerosols in the atmosphere. This paper presents an overview in the forest fire detection, emission estimation, and fire risk prediction in China using satellite imagery, climate data, and various simulation models over the past three decades. Since the 1980s, remotely-sensed data acquired by many satellites, such as NOAA/AVHRR, FY-series, MODIS, CBERS, and ENVISAT, have been widely utilized for detecting forest fire hot spots and burned areas in China. Some developed algorithms have been utilized for detecting the forest fire hot spots at a sub-pixel level. With respect to modeling the forest burning emission, a remote sensing data-driven Net Primary productivity (NPP) estimation model was developed for estimating forest biomass and fuel. In order to improve the forest fire risk modeling in China, real-time meteorological data, such as surface temperature, relative humidity, wind speed and direction, have been used as the model input for improving prediction of forest fire occurrence and its behavior. Shortwave infrared (SWIR) and near infrared (NIR) channels of satellite sensors have been employed for detecting live fuel moisture content (FMC), and the Normalized Difference Water Index (NDWI) was used for evaluating the forest vegetation condition and its moisture status.

## 1. Introduction

Forests play an important role in the global ecological, environmental and recreational functions [1,2]. It is well recognized that the forests can absorb atmospheric carbon, regulate rainfall, moderate temperature, and restrain soil erosion [3,4]. To a large extent, the forest fire is a dominant disturbance factor in almost all the forest vegetation zones throughout the World, and it is considered to be a potential hazard causing physical, biological, and environmental consequences [5–8]. Furthermore, forest fires can have adverse societal impacts regardless of whether they are caused by natural forces or human activities.

The forest resources are very limited in China, and only account for 20.36% of the whole national’s territory [9]. According to the Seventh National Forest Resources Inventory during 2004 to 2008, the forest area was estimated at 195 million ha and its stock volume was 13.72 billion cubic meters, ranking fifth in the world after Russia, Brazil, Canada, and the U.S.A [9,10]. It was reported that the forest fires burn an average of 0.86% of the total forest area every year in China [11]. Temporally, over the past 40 years, the number of forest fires has increased significantly [12]. During the period from 1950 to 1997, about 676,000 forest fires occurred and the average burned area reached about 822,000 ha each year [13]. One of the largest forest fires occurred in the Daxing’anling Forest of the Northeast China during the period from 6 May to 2 June, 1987. The burned area was in excess of 1.3 million ha of the prime forest, resulting in the loss of over 200 lives and over 50,000 homes [14]. As a result, the frequent occurrence of forest fires has become an important issue in view of the forest degradation in China [11,15–17].

Early detection and real-time monitoring of fire activities are essential for fire management and planning. Given the high cost and potential hazards associated with the ground-based and air-borne fire monitoring, space-borne satellite sensors have been widely used to detect and monitor forest fires in recent years [18–21]. Earth observations provide useful data for developing consistent method for detecting area and severity of active burning at national, continental or global scales. Sensors onboard many satellites with multi-spatial and multi-spectral resolutions, such as NOAA/AVHRR (the National Oceanic and Atmospheric Administration/the Advanced Very High Resolution Radiometer) [19,20], EOS-MODIS (Earth Observing System-Moderate Resolution Imaging Spectroradiometer) [21–24], Landsat ETM (Enhanced Thematic Mapper) [25–28] were designed to measure reflected and emitted radiation in the visible (VIS, 0.4–0.7 μm), the near infrared (NIR, 0.7–1.3 μm), the short-wave infrared (SWIR, 1.3–8 μm), the middle infrared (MIR, 3–8 μm), and the thermal infrared (TIR, 8–13 μm).

The satellite sensors such as NOAA/AVHRR, Chinese FY (FengYun)-series, MODIS, CBERS (China-Brazil Earth Resources Satellite), and ESA (European Space Agency) ENVISAT (Environmental Satellite) have been widely applied to detect forest fire hot spots and burned areas in China [29]. The relatively high temporal and spatial resolution data acquired by the polar orbiting satellites, such as NASA EOS Terra/Aqua, and Chinese FY polar orbiting series, increase the likelihood that cloud-free data can be obtained for mapping the distribution of the large-scale burned areas. In contrast, the Geostationary platforms, such as NOAA GOES (Geostationary Operational Environmental Satellite), MTSAT (Meteorological Satellite), and Chinese FY geostationary orbiting series satellites can provide continuous observations over large geographic regions, making it possible to detect the short duration fires and also to resolve the diurnal behavior of large fires [29].

Biomass burning is a major contributor of particulate matter (PM) and trace gases to the global troposphere [30,31]. Recent studies indicated that the global interannual variability in terrestrial ecosystem fluxes and in the atmospheric CO_2_, are strongly influenced by forest fire emissions [32,33]. It is noted that China’s forests have greatly contributed to the carbon sink in the global carbon budgets during the last five decades [34]. However, recent increases in forest fires in China have already caused an increase in CO_2_ emissions [35,36].

The forest fire risk prediction is very important for fire management and fire protection strategies planning. The occurrence and spread of forest fire are basically determined by the fuel property, weather conditions, and terrain features. A prerequisite for the strategy planning is the ability to identify forest fire risk zones across both broad areas and local sites. Forest fire risk zones are locations where a fire is likely to start, and from where it can easily spread to other areas [37].

In this paper, we primarily present an overview on the status of detecting forest fire hot spots and fire areas by using satellite sensors in China over the past three decades, and analyze a few developed algorithms used for detecting fires with greater accuracies. We illustrate a forest fire emission model used to estimate gas and aerosol emissions in China. We also evaluate forest fire risk potential models that have been used for predicting the forest fires occurrence in China.

## 2. Forest Fire Detection by the Satellite data in China

### 2.1. The NOAA Satellite

**Figure 1.**
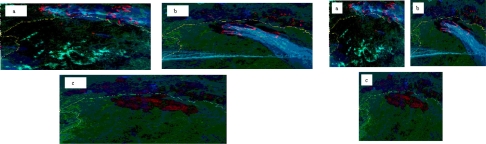
Daxing’anling forest fire hot-spot and burnt area detection by using NOAA/AVHRR.

Forest fires detection and monitoring in China have been achieved using a variety of space-borne systems/sensors in last three decades. In the early 1980s, the NOAA/AVHRR satellite images were used to detect the fire hot spots by thresholding of three infrared channels, including Ch. 3 (3.55–3.93 μm), Ch. 4 (10.5–11.3 μm) and Ch. 5 (11.5–12.5 μm), and combined BT (bright temperature) and NDVI (Normalized Difference Vegetation Index) were used for monitoring burned areas. Figure 1 represents the detected fire spot and some extensive burned areas in the Daxing’anling forest area by using NOAA/AVHRR satellite data through study by the National Satellite Meteorological Center, China [38].

Figure 1 shows the Daxing’anling forest fire hot-spot and burnt area detection by using NOAA/AVHRR satellite data during 05/06/1987 and 02/06/1987. The multi-false-color composite method with AVHRR channels 3 (3.55–3.93 μm, R), 2 (0.725–1.1 μm, G) and 1 (0.55–0.9 μm, B) was used to detect the outbreak of the forest fire in 1987/05/6 (Figure 1a); the spreading of the forest fire in 1987/05/8 (Figure 1b); and burnt area after the extinguishing fire in 1987/06/2 (Figure 1c), respectively. In the multi-false-color composite images, the red, dark red, green, and gray colors stand for active fire, burnt area, vegetation and smoke, respectively. The yellow line is border between China and Russia.

### 2.2. The Chinese Polar Orbiting Meteorological Satellite FY-1C/1D

Chinese FY-1C/D satellites have a Multi-channel Visible and Infrared Scan Radiometer (MVISR) with ten spectral channels, similar to that of the NOAA/AVHRR instrument. The channels of MVISR onboard FY-1C/D satellites are useful for detecting forest fire and burned areas (Table 1).

**Figure 2.**
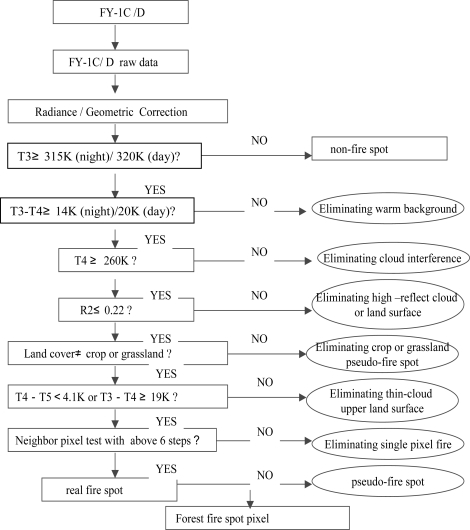
The basic flow of the forest fire detection procedure based on FY1-C/D satellites data.

Figure 2 presents a basic workflow of the forest fires detection based on the FY-1C/1D satellites data using brightness temperature and reflectance channels. The algorithm uses a combination of an absolute temperature threshold (315 K at Ch. 3, 3.55–3.95 μm) and a series of contextual tests to detect active fires by using both the Ch. 3 and Ch. 4 (10.3–11.3 μm). In addition, it incorporates warm background, cloud, land surface masking and several false alarm rejection tests. In Figure 2, the T3 and T4 stand for the bright temperature of channels 3 and 4 in FY-1C/D, respectively; and R2 is the reflectance of channel 2.

### 2.3. The Chinese Geostationary Meteorological Satellite FY-2

The polar orbiting satellites, including the NASA’s Terra and Aqua satellites, can offer a regional coverage but provide only one daytime and one nighttime observation every 24 hours. In order to develop and implement a near real-time operational fire monitoring network for China, the China Meteorological Administration processed and analyzed imagery acquired from the China FY-2C/D, together with imagery from the GOES, MTSAT geostationary satellites. The China FY-2 series geostationary satellites are operationally similar to the GMS (Geosynchronous Meteorological Satellite) with high-resolution stretched VISSR (Visible and Infrared Spin Scan Radiometer) data [39]. The channel characteristics of VISSR onboard FY-2C/2D satellites are shown in Table 2.

**Figure 3.**
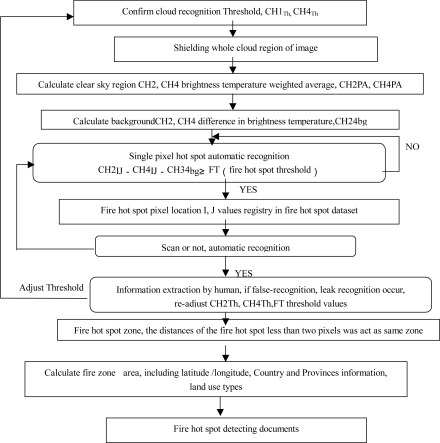
Forest fire hot spot detecting flow chart using FY-2C/2D satellites data.

**Figure 4.**
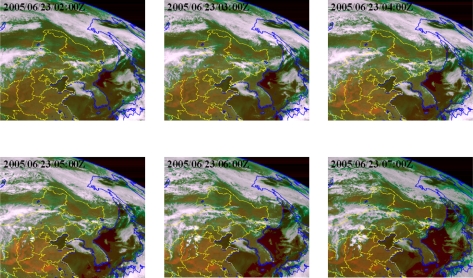
Forest fires hot spot monitoring hourly by using FY-2C satellite data.

A complete 20° × 20° scan covering the full earth disk can be accomplished each 30 minutes. Most forest fires have strong diurnal cycles, and in some regions the fires are short-lived lasting no more than a couple of hours. The geostationary satellite provides frequent and timely acquisitions to allow detection and monitoring of active fires from space. Figure 3 illustrates workflow of the forest fire hot spot detection using FY-2C/2D satellites data. Figure 4 shows the hourly progress of the forest fires hot spot obtained by using the FY-2C imagery.

Figure 3 shows the forest fire hot spot detecting flow chart using FY-2C/2D satellites data. The Ch. 1_Th_, Ch. 4_Th_ are the bright temperature (BT) threshold of Ch. 1 and Ch. 4; CH2PA, CH4PA are the BT weighted average of Ch. 2 and Ch. 4; Ch. 24_bg_ are the blackguard BT difference of Ch. 2 and Ch. 4; Ch. 2_IJ_, Ch. 4 _IJ_ are the BT of Ch. 2 and Ch. 4 at I, J pixel location. FT is the fire hot spot threshold.

Figure 4 shows the hourly distribution of forest fires detected (red spots) and observed land surface (yellow signs) located across the Northeast China and Russia during 02:00 to 07:00 (Beijing time) on 23 July, 2005 based on FY-2C satellite forest fire detected method (see Figure 3). The background is an image of land cover types and water bodies.

### 2.4. The CBERS: China-Brazil Earth Resources Satellite

The CBERS was jointly developed by China and Brazil. The Payloads of CBERS-1/2 have three sensors: a Charge Coupled Device Camera (CCD), an Infrared Multi-Spectral Scanner (IRMSS), and a Wide Field Imager (WFI). Table 3 summarizes the CBERS-1/2 payloads, sensors and channels characteristics [40]. The CCD camera has a nadir spatial resolution of 19.5 m and a swath width of 113 km with four spectral bands in the VIS and NIR ranges and one panchromatic band. The CBERS IRMSS has a spatial resolution of 78 m (for three VIS and SWIR bands) and 156 m (for one TIR band). The swath width is 119.5 km. One purpose of the CBERS is to facilitate detection of fires with relatively high spatial resolution. Currently, the forest fire detection information system based on the CBERS data has been established in China [41]. The burned areas can be detected by using the CBERS-02 satellite data of CCD Ch. 4 (0.77–0.89 μm, Red), Ch. 3 (0.63–0.69 μm, Green), and Ch. 2 (0.52–0.59 μm, Blue).

### 2.5. The EOS/MODIS Satellite

A key instrument of the NASA’s EOS Terra and Aqua missions, the MODIS was designed to measure the reflected and emitted radiation in multiple channels including VIS, NIR, MIR, and TIR.

**Figure 5.**
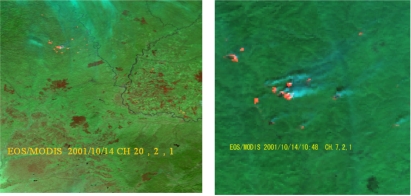
Forest fire hot spot detection using EOS/MODIS in Northern China.

The MODIS data provide good temporal coverage (four time daily), high spectral resolution (36 bands total) and a medium spatial resolution (250 m, 500 m, and 1000 m). The MODIS has a higher spatial resolution (250 m) than that of AVHRR for Band 1 (0.620–0.670 μm) and Band 2 (0.841–0.876 μm). Additionally, the images and high level products are freely accessible. The four time daily MODIS images greatly enhanced fire monitoring on a global scale with a 1 km spatial resolution for MODIS channels at 4 μm (Ch. 21, Ch. 22) with high saturation level at 500K and 331K, and 11 μm (Ch. 31) with high saturation level at approximately 400 K for the Terra MODIS and 340K for the Aqua MODIS, respectively [22,42,43]. The MODIS Ch. 1 and Ch. 2 are usually applied to detect the burned areas and the smoke; and the Ch. 1, Ch. 2 Ch. 6, Ch. 7, Ch. 20– Ch. 25, Ch. 31 and Ch. 32 are used to detect active fire [22,29]. The MODIS active fire algorithm was developed by NASA [22,42], and have been used in detecting forest fire in many regions in the world [43–47].Chinese researchers began to detect forest active fires using MODIS active fire algorithm developed by Kaufman *et al.* from 2001 [48–51], and forest active fire can be detected accurately in different region of China, except that a few low temperature hot spots might be missed, depending on the differences of regions or seasons [52]. Zhou *et al.* reported that the MODIS band 7 is sensitive to high temperature, not sensitive to medium temperature, and this can be used to verify fire intensity [53]. We developed a MODIS three channels active fire algorithm. Figure 5 shows fire hot spots detected using the EOS/MODIS data in Northern China. In this case, we used Ch. 20 (3.66–3.84 μm), Ch. 2 (0.84–0.87 μm) and Ch. 1 (0.62–0.67 μm) (shown in the left image of Figure 5) and Ch. 7 (2.10–2.13 μm), Ch. 2 and Ch. 1 (shown in the right image of Figure 5) to form some false-color composites to detect forest fire hot spots and smoke plumes.

Figure 5 presents an example of forest fire hot spot detection using the EOS/MODIS in Northern China on 14 October, 2001. The left image was mapped using MODIS Ch. 20 (3.66–3.84 μm, R), Ch. 2 (0.84–0.87 μm, G) and Ch. 1 (0.62–0.67 μm, B); and right image was mapped using MODIS Ch. 7 (2.10–2.13 μm, R), Ch. 2 (G) and Ch. 1 (B) through the false-color composite analysis to detect forest fire hot spot (red colour) and smoke plumes. For MODIS, the smoke can be much more easily identified.

### 2.6. The ESA ENVISAT Satellite

The ESA satellite, ENVISAT, was launched in 2002 with an expected lifespan of five years [54]. The ENVISAT satellite provides both optical and radar measurements of the Earth’s surface. Three ENVISAT instruments can be used to investigate the extent and impact of the forest and peatland fire. Reduced spatial resolution Medium Resolution Imaging Spectrometer (MERIS) imagery was used to identify simple land cover features and smoke plumes. The fire hot spots were detected by band 3.7 μm of Advanced Along Track Scanning Radiometer (AATSR) nighttime acquisitions, and burned areas were detected by Advanced Synthetic Aperture Radar (ASAR) wide swath radar imagery acquired before and after the fire events. The performance and the capability of the ENVISAT to acquire data from different sensors simultaneously or within a short period of time greatly enhance the possibilities to monitor forest fires occurrence [55,56].

Under the Dragon Program sponsored by the ESA and the Ministry of Science and Technology of China, the studies have been carried out in detecting forest fires using AATSR/ENVISAT data [16,57,58], and extracting edges of burned region using MERIS/ENVISAT data. Validation of the algorithms has been made by using ground fire information and detected forest fire hot spot obtained from the MODIS data. It is found that the edges of burned region can be efficiently extracted from the MERIS images through image enhancement technique and by human-machine interactive interpretation [16,58].

## 3. New Techniques Used in Detection of Forest Fire in China

### 3.1. Estimation of Sub-Pixel Fire Burned Areas and Temperature

Many algorithms developed for detecting forest fire use a mid-infrared band (3.5–4.0 μm) and a thermal band (10.5–12.5 μm) [19–24]. The burning objects are separated from the background by various image processing algorithms. However, the spatial resolution of the infrared channels in the majority of the meteorological and environmental satellites is no finer than 1 km; it limits the ability to accurately quantify spatial extent of the fires, because the actual size of many burned areas can be less than 1 km^2^, especially in the beginning of the fire occurrence. Liu *et al.* developed two kind of methods for estimating sub-pixel forest fire burned areas and temperature using the multiple infrared channels from NOAA/AVHRR data [59]. One method uses both MIR band (Ch. 3, 3.7 μm) and TIR band (Ch. 4, 11 μm). A set of non-linear equations were established for the mixed pixels using different infrared bands, and a Newton’s iteration method was used to estimate forest fire burned areas and associated temperature at sub-pixel scale. The other method uses two TIR bands (Ch. 4 and Ch. 5, 11 and 12 μm), and an equation of BT increment in Ch. 4, and another equation of BT increment differences in Ch. 4 and Ch. 5 of all mixed pixels were established. Finally, by using a Look-Up Table (LUT), the fire burned areas and associated temperature can be retrieved at sub-pixel scale [59].

### 3.2. Auto-Identification of Forest Fire Hot Spots

The traditional approach to identify forest fire hot spots often involves visual interpretation of satellite images. There is a need to develop techniques for auto-identifying forest fires spots, so that fires can be detected and managed in timely manner. The artificial neural network (ANN) theory with Back Propagation (BP) learning algorithm provides a new means to solve this problem [60]. Liang *et al.* developed an ANN method to detect forest fire spots in Hubei Province of China using 43 meteorological satellite images [61]. Out of these images, 31 were used for collecting training samples and the rest were used for assigning testing samples. The results showed that the identification of the fire spot was related to seven characteristics associated with the radiation values of the five channels of the NOAA/AVHRR, the land cover types, and the differences of contiguous BT. The ANN method can capture the major characteristics of fire spots and non-fire spots, and has an ability to automatically identify fire spots from the satellite images [61]. Compared to the visual interpretation, the ANN method can identify fire hot spot from the satellite images with a similar accuracy [60–63]. Maeda *et al.* [64] also used MODIS imagery and ANN method to predict forest fire in the Brazilian Amazon. The result showed that the spatial distribution of the areas with fire risk were consistent with the fire events observed. The ANN model allowed a fast and relatively precise method to predict forest fire events in the studied area [64].

### 3.3. Establishment of a New Fire Detection Channel Selection from Fire Experiment

For the purpose of selecting the most effective spectral channels for identifying forest fires, a controlled experiment was carried out at a designated place in a pine-dominated forest site in Wuming County, Guangxi Province, China in October 2005 [65]. The infrared and visible spectral radiances and BT were measured synchronously at the time of satellite overpass using a medium and near-infrared MOMEM MR154 FT-Spectroradiometer, an infrared thermal imager, and a visible and near-infrared spectroradiometer (ASD FR). The results showed that two radiance peaks in the MIR band corresponded to the fire burning strength. One peak at 4.17 μm is located at the CO emission peak [66]. The other peak spans through the wavelengths of 4.34–4.76 μm, which exhibited a stronger response to the fire than the commonly used wavelengths of 3.5–4.0 μm, and seem to be more sensitive band for the fire detection using remote sensing techniques [65].

### 3.4. A New Algorithm for Fire Burned Areas Identification

Many techniques use a set of thresholds based on spectral bands together with a time series analysis to detect rapid and dramatic land cover changes in order to monitor burned areas [67]. Some algorithms developed in the past decade included Glo bal Environment Monitoring Index (GEMI) [68,69]; VI3T [70]; Shortwave Vegetation Index (SWVI) [71], Burn Area Index (BAI) [72], Normalized Burn Ratio (NBR) [73]. Based on the GEMI and spectral characteristics of burned areas in the short wave region, Tan *et al.* developed an adjusted GEMI-Burned areas (GEMI-B) method to accurately extract the fire burned areas in grasslands of China by using medium-resolution EOS-MODIS satellite imagery with channels 5 and 7 [74] An automated extraction procedure was also developed using active fire (hot spot) as seeds and GEMI-B image. The GEMI-B algorithm was used to detect fire occurrence in the border areas between China and Mongolia on 21–31 May, 2003. The result showed that the burned areas extracted by the new method were consistent with that from the high resolution Landsat TM data [74].

## 4. Forest Fire Emissions Estimation in China

**Figure 6.**
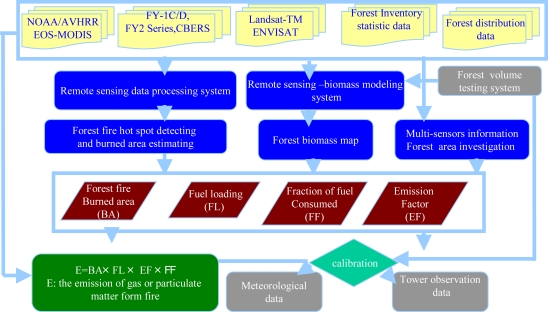
Forest fire emissions estimation system in China.

Biomass burning emissions including greenhouse gases such as CO_2_, CH_4_ and aerosol particles have significant influences on air quality, atmospheric chemical composition, the earth’s radiation budget, and global climate change [75–79]. In recent years, the forest fires have significantly aggravated and affected national forest ecosystem due to human activities and some extreme climate events. The forest fires are believed to have generated much carbon emissions in China [35,36]. An approach for addressing biomass burning emissions is to accurately quantify the amount of emissions spatially and temporally based on observations of active fires. Figure 6 presents a flowchart of the Chinese Forest Fire Emission Estimation System (named CFFEES).

Figure 6 shows the forest fire emissions estimation system in China. In the CFFEES, the forest fire burned area (BA) and fuel loading (FL) detection by using multi-sensors including NOAA/AVHRR, FY-series, EOS-MODIS, Landsat-TM, ENVISAT *etc.*; and which are integrated with forest type (or land use), forest inventory data, the fraction of fuel consumed (FF) in live vegetation data, and the associated emission factor (EF). The CFFEES may calculate the forest fire emissions of various gases (*i.e.*, CO_2_, CO, CH_4_) or PM by using the emission model showed in Figure 6. All the source data are spatially and temporally distributed and daily assimilated according to the forest burning hot spot defined by using the satellite observations. The entire estimated emissions of forest fire using the CFFEES system can be calibrated by using the flux tower observation data.

### 4.1. Forest Biomass Simulation Based on BEPS Model and Satellite data

In the CFFEES model, it is important to have an accurate estimation of forest biomass (or fuel loading). Brown (1984) started to estimate biomass of a tropical forests based on the forest volumes [80]. Recently, many bio-ecological models using remote sensing data as input have been used to estimate the net primary productivity (NPP) and biomass [81–85]. Liu *et al.* developed a boreal ecosystems productivity simulator (BEPS) model based on the Forest BioGeochemical Cycles (FOREST-BGC) model for quantifying the biophysical processes governing ecosystems productivity [81]. The BEPS model has been used to accurately estimate forest NPP in two forest sites of Northeast China and one forest site in Western China when using the remotely sensed data of high spatial resolution as input. The results show that the observed NPP agrees well with the modeled NPP from BEPS [86,87]. Others examples in China also support that the estimated forest NPP are highly related to field data in the study area by using BEPS model [88–91].

### 4.2. Quantifying Emission of Forest Fire in China using Satellite Data and Emission Model

Forest fire is a major process and contributes to an increasing concentration of pollutants in the atmosphere, such as CO_2_ CO, CH_4_, and aerosols in China [35,36]. Tian *et al.* estimated the amount of carbon emissions directly from forest fires in China during 1991 to 2000 using satellite data and an emission model [35]. It was concluded that during this period China’s forest fires emitted CO_2_ 74.2–104.7 Tg, CH_4_ 1.797–2.536 Tg, and smoke aerosols 0.999–1.410 Tg. During 1991–2000, the average CO_2_ and CH_4_ emissions of forest fire accounted for 2.7%–3.9% and 3.3%–4.7% of the total CO_2_ and CH_4_ emissions, respectively.

For examining the spatial and temporal patterns of the fire-induced carbon emissions, Lü *et al.* [36] estimated the emission of carbon (C) and carbon-containing trace gases including CO_2_, CO, CH_4_, and nonmethane hydrocarbons (NMHC) from forest fires in China from 1950 to 2000 by using a combination of remote Sensing (Landsat TM/ETM images) [92], the forest fire inventory, and a terrestrial ecosystem model. The results showed that mean annual carbon emission was about 11.31 Tg from forest fires in China, ranging from a minimum of 8.55 Tg to a maximum of 13.9 Tg. This amount of carbon emission resulted from the atmospheric emissions of four trace gases, *i.e.*, 40.66 Tg CO_2_ with a range from 29.21 to 47.53 Tg; 2.71 Tg CO with a range from 1.48 to 4.30 Tg; 0.112 Tg CH_4_ with a range from 0.06 to 0.2 Tg, and 0.113 Tg NMHC with a range from 0.05 to 0.19 Tg [36]. Wang *et al.* [93] studied the influence of fire on carbon distribution and net primary production of boreal forests in north-eastern China, the result showed that the 1987 conflagration in north-east China released 25–49 Tg C to the atmosphere. Information on the amount of emission by forest fires should help the investigation on the transportation and dispersion studies of the plumes, and also provide important input data to the climate models.

## 5. Forest Fire Risk Prediction in China

The forest fire risk prediction is important for fire management and planning; in particular, forest fires are known by distinct spatial and temporal characteristics in China.

### 5.1. The Fire Spread Behavior Model

Forest fire spread is basically determined by the fuel property, meteorological factors and terrain. The process of forest fire spread contains the self-replicating feature, and forest fire behavior can be described by the fractal geometry. Zhu *et al.* simulated the fire spread based on remotely-sensed data (Landsat TM and AVHRR imagery) and a geographic information system [94]. A method of limited spreading lumping was adopted to describe the growing phenomena and to simulate the dynamic process of the forest fire spread. The result of fire simulation was adjusted by a scale rule. The simulated fire and the actual fire manifest the self-similarity in their spreading shapes as well as the quantitative similarity in their areas.

Malamud *et al.* indicated that forest fire spread is an example of self-organized criticality (SOC) behavior [95]. Song *et al.* improved the traditional forest-fire spread cellular-automata model, and considered the effect of tree species, meteorological conditions and human activities on the forest fire spread [96]. It was concluded that forest fires occurrence and spread showed a significant SOC behavior in China for the period from 1950 to 1989, and simulation result using the improved model was better with the actual forest fire occurrence and spread data than those of the traditional model.

### 5.2. Forest Fire Risk Prediction Based on Satellite Data and GIS

A forest fire potential model using satellite data and GIS (Geographic Information System) was developed to enhance appropriate fire management plans through identifying areas of high possibility for fire outbreak in China. In the fire risk model framework, the input data includes land cover map, road and village map, forest management map, vegetation water content map, and DEM (Digital Elevation Model) map. Output products include fuel risk sub-model map, fire detection sub-model map, fire response sub-model map and final fire risk model map. For instance, Xu *et al.* used remotely-sensed data and GIS to map the forest fire potential of Baihe forestry bureau in Jilin Province [97]. The satellite images were interpreted and classified to generate vegetation type layer and land use layers (roads, settlements and farmlands); topographic layers (slope, aspect and altitude) were derived from DEM. The forest fire risk zones were delineated by assigning subjective weights to the classes of all the layers (vegetation type, slope, aspect, altitude and distance from roads, farmlands and settlements) according to their sensitivities to the fire or their fire-inducing capability. The mapping results of the study area were found to be in strong agreement with the actual fire-affected sites.

The principle component analysis (PCA) method is often used to sort out the relationships between forest fire potential and environmental factors. The classifications of the factors generated from the PCA analysis were performed with GIS to generate three maps: a fuel-based fire risk map, a topography-based fire risk map, and an anthropogenic-factor fire risk map. These three maps were then synthesized to generate the final fire risk map. A linear regression method was used to analyze the relationship between an area-weighted value of forest fire risks and the frequency of historical forest fires at each forest farm[98]; the results show that the most important factor contributing to forest fire occurrence is topography, followed by anthropogenic factors.

### 5.3. Forest Fuel Moisture Content Estimation Model

The estimation of forest fuel moisture content (FMC) is important for predicting forest fire danger. With use of remote sensing sensors, it is possible to retrieve vegetation water content. Several studies demonstrated the linkage between leaf-level reflectance in the 400–2500 nm spectral region and the FMC [99–102]. A weak absorption in the 950–970 nm wavelength range was found to be useful for detecting the water status of vegetation and FMC [99]. The spectral features in reflectance spectra of green vegetation in 1300–2500 nm regions are mainly dominated by the liquid water absorption [100]. Gao *et al.* calculated the normalized difference water index (NDWI) as (R860–R1240)/(R860+R1240) in a theoretical study and the results demonstrated that the index has potential applicability for FMC detection [101]. The AVIRIS (Airborne Visible/Infrared Imaging Spectrometer) imagery also was used to study the possibility of retrieving FMC [103,104]. The NDWI (860, 1250) derived from EOS/MODIS data has been used to estimate FMC (*i.e.*, EWT, equivalent water thickness) in the northern and eastern China in July 2006, which showed a potential of detecting FMC on a regional scale [105].

### 5.4. Comparison between the Fire Risk Rating Systems in China and Those Used in Other Regions

Several countries including Australia, Canada and U.S.A. have developed sophisticated Forest Fire Danger Rating Systems (FFDRS) [106–112]. The U.S. National Fire Danger Rating System (NFDRS) was constructed in 1968. In the NFDRS, a Fire Danger Rating level takes into account current and antecedent weather, fuel types, and both live and dead fuel moisture [107–109]. Existing large fire forecast products are utilized, specifically Relative Greenness (RG), the Fire Potential Index (FPI), and 7-day weather forecasts from the National Digital Forecast Database (NDFD) to produce 7-daily forecast maps of FPI, plus four large fire probability maps, and a table that presents the probability of large fires by Geographic Area Coordination Center (GACC) areas of responsibility [110].

The Canadian Forest Fire Danger Rating System (CFFDRS) has been under development since 1968. Currently, two subsystems–the Canadian Forest Fire Weather Index (FWI) System and the Canadian Forest Fire Behavior Prediction (FBP) System—are being used extensively in Canada and internationally. The components of the CFFDRS include risk, weather, fuels, and topography, and provide the necessary inputs to predict fire weather, fire occurrence, and fire behavior. Fuel moisture models are currently being developed for a range of Canadian forest types. Together, these systems predict the potential fire danger within the forest [111,112].

While in China, above-mentioned systems such as FFDRS and CFFDRS are still difficult to implement, because they are based on a lot of heterogeneous sources data, such as fuel property and fire characteristics data from in situ or remote sensing observations, meteorological data from observations and model prediction, topography data over long temporal scales but high spatial resolution, and socio-economic data.

In order to support the decision making processes for Chinese government, a national forest fire risk weather danger has been promulgated [113], and a forest fire risk rating system at national-level has been developed [114,115]. The system selects a prediction model according to forest fire risk rating and fire risk indexes defined by well-established standards in forestry. The system obtains daily meteorological dataset and information on weather forecast, and observed data of fire risk factors collected (such as fuel growing information, fuel water content, fuel types *etc.*) from some key areas. It then generates the forest fire risk prediction for future 24 and 48 hours. The forest fire risk prediction results are delivered daily through a special forestry network and are published 4 times a week through a China Central Television Program. The forest fire risk monitoring operation has been very beneficial to China for detecting the fast changing burned areas, particularly when fires occur over the remote and sparsely populated areas.

## 6. Conclusions and Remarks

This paper has provided an overview of the fire detection, emission estimation and risk prediction via operational satellite sensors, simulated models and climate information in China in the past three decades. Overall, in the last 30 years, China has developed its meteorological and environmental satellite sensors to detect forest fires across the nation and the surrounding areas. The second generation of China’s polar orbiting meteorological satellite (Fengyun-3) with substantially advanced multi-spectral imaging systems was launched on 28 May, 2008. The disaster and environment monitoring and forecast small satellite constellation was launched on 6 September, 2008, and comprised two satellites (HJ-1A and HJ-1B). There are three kinds of optical remote-sensing sensors on board, multi-spectral imager (CCD) with 360 km swath and 30 m spatial resolution, infrared imager (IRS) with 720 km swath and 150 m spatial resolution, and hyper-spectral imager (HIS) with 50 km swath and 100 m spatial resolution, and 110 channels range 0.45–0.90 μm [116]. The CCD (0.43–0.9 μm) and IRS data (3.5–3.9 μm) work well for the hot-spot detection and forest fires assessment [116–118]. Along with other currently operational satellites such as NOAA/AVHRR, FY-series, EOS/MODIS, ENVISAT and CBERS, a more accurate and efficient operational system in fire detection, emission estimation and risk prediction can be established and operated before, during and after a fire season. However, for the fire risk management one needs to take into account and assess the fire risk potentials.

A forest fire can be a real ecological disaster regardless of whether it is caused by natural forces or human activities. It is possible to map forest fire risk zones to minimize the frequency of fires and avert damage. As climate changes and extreme climatic events become more prevalent, the frequency and intensity of forest fire occurrence will likely increase in China and the surrounding areas. It is important to develop a new fire danger and risk model to forecast the forest fire potential. Accurate forecasting of the El Niño event occurrence is critical.

Forest fires have significant impact on vegetation dynamics and deplete timber resources, so that forest fires monitoring is a critical aspect of sustainable forest management. Forest fires have direct and important effects on the global carbon cycle, atmospheric chemistry, regional radiation and climate and in regulating terrestrial ecosystems and biodiversity [119]. Uncertainty over the effects of future climate change upon fire regimes, and the importance of vegetation-atmosphere feedbacks has fostered increased effort to develop coupled models of vegetation and fire to understand these future changes. To reach a closure in carbon balance including some greenhouse gases as CO_2_ and CH_4_, *etc.*, and to understand smoke aerosols produced from burning and which influence global climate and atmospheric chemistry, the forest fire emissions estimates based on satellite sensors is very important and complex. Additional studies should be carried out to accurately detect forest burned areas, fragmentation of burned scars, fuel loading and biomass based on the multi-sensors. Further investigation, analysis, and computations of the fraction of fuel consumed by forest fire, and emission factors for the different vegetation types are necessary to quantitatively evaluate and verify Chinese forest fires emissions in the future.

## Figures and Tables

**Table 1.** The channel characteristics for detecting forest fire by using MVISR sensor onboard FY-1C/1D satellite.

**Channel**	**Wavelength (μm)**	**Resolution (km)**	**Dynamic range**	**Detecting sensitivity**	**Fire detection**
1	0.58–0.68	1.1	0–90%	S/N ≥ 3 (ρ = 0.5%)	Burnt area
2	0.84–0.89	1.1	0–90%	S/N ≥ 3 (ρ = 0.5%)	Burnt area
3	3.55–3.95	1.1	190–340 K	NEΔ*T* ≤ 0.4 K (300 K)	Hot-spot
4	10.3–11.3	1.1	190–330 K	NEΔ*T* ≤ 0.22 K (300 K)	Hot-spot
5	11.5–12.5	1.1	190–330 K	NEΔ*T* ≤ 0.22 K (300 K)	Burnt area

S/N: signal to noise ratio; ρ: reflectivity; NEΔ*T*: Noise-Equivalent Temperature Difference.

**Table 2.** The channel characteristics of VISSR onboard FY-2C/2D satellites.

**Channel**	**Wavelength (μm)**	**Resolution (km)**	**Dynamic range**	**Temperature resolution (K)**	**S/N**	**Primary use**
1	0.5–0.9	1.25	0–98%		0.5ρ = 2.5% 95ρ = 95%	Burnt area
2	3.5–4.0	5	180–330 K	0.6–0.5		Hot-spot
3	6.3–7.6	5	180–280 K	0.5–0.3		Water vapor
4	10.3–11.3	5	180–330 K	0.4–0.2		Hot-spot
5	11.5–12.5	5	180–330 K	0.4–0.2		Burnt area

ρ: reflectivity; S/N: signal to noise ratio.

**Table 3.** The CBERS payloads, sensors and channels characteristics.

**Payload**	**CCD**	**IRMSS**	**WFI**
Sensor Type	Push-broom	Electro-mechanic	Push-broom
Visible and near infrared bands (μm)	1: 0.45–0.52 2: 0.52–0.59 3: 0.63–0.69 4: 0.77–0.89 5: 0.51–0.73	6: 0.50–0.90	10: 0.63–0.69 11: 0.77–0.89
Shortwave infrared bands (μm)		7: 1.55–1.75 8: 2.08–2.35	
Thermal infrared bands (μm)		9: 10.4–12.5	
Resolution (m)	19.5	Band 6–8: 78 Band 9: 156	258
View angle	8.32°	8.80°	59.6°
Swath wide (km)	113	119.5	890

CCD: Charge Coupled Device Camera; IRMSS: Infrared Multi-Spectral Scanner; WFI: Wide Field Imager.
